# Whole-Genome Sequence of Haloimpatiens lingqiaonensis Strain P8956

**DOI:** 10.1128/MRA.00699-19

**Published:** 2019-10-24

**Authors:** Hussein Anani, Didier Raoult, Pierre-Edouard Fournier

**Affiliations:** a Aix Marseille Université, Institut de Recherche pour le Développement (IRD), Service de Santé des Armées, AP-HM, UMR Vecteurs Infections Tropicales et Méditerranéennes (VITROME), Institut Hospitalo-Universitaire Méditerranée Infection, Marseille, France; b Institut Méditerranée-Infection, Marseille, France; c Aix-Marseille Université, Institut de Recherche pour le Développement (IRD), UMR Microbes Evolution Phylogeny and Infections (MEPHI), Institut Hospitalo-Universitaire Méditerranée-Infection, Marseille, France; d Special Infectious Agents Unit, King Fahd Medical Research Center, King Abdulaziz University, Jeddah, Saudi Arabia; University of Maryland School of Medicine

## Abstract

In 2016, Haloimpatiens lingqiaonensis was described as a bacterial isolate from paper mill wastewater. Previously, no whole-genome sequence was available for this microorganism. Whole-genome sequencing of strain P8956 yielded a 3,295,388-bp genome with a 30.7% G+C content, 2,917 protein-coding genes, and 95 predicted RNA genes.

## ANNOUNCEMENT

In 2016, Wu et al. proposed the creation of the bacterial genus *Haloimpatiens* within the family *Clostridiaceae* and the phylum *Firmicutes* (1). Strain ZC-CMC3^T^ was described as the type strain of the species Haloimpatiens lingqiaonensis (2). Strain ZC-CMC3^T^ had been isolated from wastewater samples collected from a paper mill in Lingqiao City, Zhejiang, China (2). The organism was isolated as described by Wu et al. in 2016 (1). In our laboratory, growth of Haloimpatiens lingqiaonensis strain P8956 was obtained after 24 h of culture in 5% sheep blood-enriched Columbia agar (bioMérieux, Marcy l’Etoile, France) in an anaerobic atmosphere at 37°C (3–5). This bacterium exhibited a 94.04% 16S rRNA sequence similarity with Hathewaya histolytica strain JCM 1403^T^ (GenBank accession number NR_113187), its closest phylogenetic neighbor.

In the present study, DNA from Haloimpatiens lingqiaonensis strain P8956 was extracted using the EZ1 biorobot and the EZ1 DNA tissue kit (Qiagen, Hilden, Germany). Extracted DNA was quantified at 0.2 μg/μl using a Qubit assay with a high-sensitivity kit (Life Technologies, Carlsbad, CA, USA) and sequenced with the MiSeq sequencer (Illumina, Inc., San Diego, CA, USA). To prepare the paired-end library, dilution was performed to require 1 ng of each genome as input to prepare the paired-end library. The “tagmentation” step fragmented and tagged the DNA. The DNA was fragmented and amplified by limited PCR (12 cycles), introducing dual-index barcodes and sequencing adapters. After purification on AMPure XP beads (Beckman Coulter, Inc., Fullerton, CA, USA), libraries were normalized and pooled for sequencing following the MiSeq System Denature and Dilute Libraries Guide 15039740-10 (Illumina kit). Paired-end sequencing and automated cluster generation with dual-indexed 2 × 250-bp reads were performed during a 39-hour run. Total information of 8.2 Gb was obtained from a 1,207,000/mm^2^ cluster density with a cluster passing quality control filters of 89.3% (10,507.2 passed filtered reads). A total of 5,876,657 reads were quality checked using FastQC and trimmed using Trimmomatic version 0.36.6 (6). MiSeq reads were assembled using SPAdes version 3.5.0 software (7). The “careful” option was used in order to reduce the number of mismatches and short indels. Default parameters were applied here and for all software (for k values, i.e., k-mer values of 127, 99, 77, 55, 33, and 21). SSPACE (8) and GapFiller (9) were used to combine contigs with default parameters. The draft genome sequence of Haloimpatiens lingqiaonensis strain P8956 is composed of 185 contigs (*N*_50_, 36,473 contigs; *L*_50_, 30 contigs; coverage, 35×) for a total of 3,295,388-bp with a 30.7% G+C content. Annotation using Prokka version 1.13 (10) predicted 3,012 genes and 2,917 protein-coding genes, 1,360 (45.15%) of which were assigned to clusters of orthologous group categories. In addition, 95 RNA genes were detected (10 rRNAs and 85 tRNAs). Using BLAST methods against resistance and toxin-antitoxin databases (11–13) with default parameters, genes with identity above 80% were taken into account. Six antibiotic resistance-associated genes were predicted, coding resistance to lincosamides (3 genes), oxacillin (1), beta-lactams (1), and mupirocin (1). No toxin-antitoxin module or bacteriocin-associated gene could be found.

### Data availability.

The draft genome and read sequences of *H. lingqiaonensis* strain P8956 (BioProject number PRJEB32392 and BioSample number SAMEA5587607) have been deposited at EBI/GenBank under the accession numbers CABDWS010000001 to CABDWS010000185 and ERR3393035.
